# Identification of a Putative Crf Splice Variant and Generation of Recombinant Antibodies for the Specific Detection of *Aspergillus fumigatus*

**DOI:** 10.1371/journal.pone.0006625

**Published:** 2009-08-13

**Authors:** Mark Schütte, Philippe Thullier, Thibaut Pelat, Xenia Wezler, Philip Rosenstock, Dominik Hinz, Martina Inga Kirsch, Mike Hasenberg, Ronald Frank, Thomas Schirrmann, Matthias Gunzer, Michael Hust, Stefan Dübel

**Affiliations:** 1 Technische Universität Braunschweig, Institut für Biochemie und Biotechnologie, Braunschweig, Germany; 2 Groupe de biotechnologie des anticorps, Département de biologie des agents transmissibles, Centre de Recherche du Service de Santé des Armées, La Tronche, France; 3 Institute of Molecular and Clinical Immunology, Otto-von-Guericke University Magdeburg, Magdeburg, Germany; 4 Helmholtz-Centre for Infection Research, Braunschweig, Germany; University of Toronto, Canada

## Abstract

**Background:**

*Aspergillus fumigatus* is a common airborne fungal pathogen for humans. It frequently causes an invasive aspergillosis (IA) in immunocompromised patients with poor prognosis. Potent antifungal drugs are very expensive and cause serious adverse effects. Their correct application requires an early and specific diagnosis of IA, which is still not properly achievable. This work aims to a specific detection of *A. fumigatus* by immunofluorescence and the generation of recombinant antibodies for the detection of *A. fumigatus* by ELISA.

**Results:**

The *A. fumigatus* antigen Crf2 was isolated from a human patient with proven IA. It is a novel variant of a group of surface proteins (Crf1, Asp f9, Asp f16) which belong to the glycosylhydrolase family. Single chain fragment variables (scFvs) were obtained by phage display from a human naive antibody gene library and an immune antibody gene library generated from a macaque immunized with recombinant Crf2. Two different selection strategies were performed and shown to influence the selection of scFvs recognizing the Crf2 antigen in its native conformation. Using these antibodies, Crf2 was localized in growing hyphae of *A. fumigatus* but not in spores. In addition, the antibodies allowed differentiation between *A. fumigatus* and related *Aspergillus* species or *Candida albicans* by immunofluorescence microscopy. The scFv antibody clones were further characterized for their affinity, the nature of their epitope, their serum stability and their detection limit of Crf2 in human serum.

**Conclusion:**

Crf2 and the corresponding recombinant antibodies offer a novel approach for the early diagnostics of IA caused by *A. fumigatus*.

## Introduction

*Aspergillus fumigatus* is a common airborne human fungal pathogen. In addition to allergic diseases *A. fumigatus* causes the highly lethal form of invasive aspergillosis (IA) [1]. In the past two decades the number of IA cases increased, due to the ever increasing number of susceptible patients [2]–[5]. The largest group among these are individuals with hematopoietic stem cell transplantation (HSCT) or solid organ transplantation requiring permanent immunosuppression [3], [6], [7]. Today, IA is the number one cause of death due to infectious complications in allogeneic bone marrow transplantation [8], despite the availability of potent drugs such as amphotericin B, azole derivatives or echinocandins [3], [9]. A possible reason for this is the gradual development of resistance in *Aspergillus* as well as the occurrence of side effects of drug usage and lack of initial response that could lead to the interruption of the treatment [10], [11]. Other diseases caused by *A. fumigatus* are the aspergilloma [12], [13] and allergic bronchopulmonary aspergillosis (ABPA) [14]–[16].

The non invasive early diagnosis of IA is currently done by real time PCR amplifying *A. fumigatus* specific DNA sequences, by enzyme-linked immunosorbent assay (ELISA) for the detection of galactomannan (GM) or an assay for the detection of (1→3)-β-D-Glucan (BG). These assays lack sensitivity and specificity, but the reliability of IA diagnosis can be improved by combining the galactomannan ELISA and PCR [17], [18], [5]. An early diagnosis of IA is critical for a successful antifungal treatment with antimycotics [19], [2]. In later stages of the IA the disease can be detected by computed tomography (CT) [20], [21].

Todate, many *A. fumigatus* specific antigens were described [1], but only a few were further characterized. Very well characterized are the *A. fumigatus* glycosylhydrolases/glycosyltranferases Asp f9, Asp f16 and Crf1. All three proteins are encoded by the *crf1* gene and are splice variants of its pre-mRNA, resulting in three different mRNAs *crf1*, *asp f9* and *asp f16*. Their proposed tranlation products have a glycosylphosphatidylinositol (GPI) anchor and are located in the cell wall of growing hyphae [22]–[27]. Asp f16 was succesfully used for active vaccination of mammals resulting in the protection of *A. fumigatus* infection [23], [28], [24], but recently the existence of Asp f16 became doubtful [29].

The aim of this study was the cloning and expression of Asp f9, Asp f16 or Crf for the generation of recombinant antibodies by antibody phage display to develop a histopathological detection system for *A. fumigatus* by immunofluorescence and a serum diagnostic assay by ELISA. In this process, a new variant of these glycosylhydrolases was isolated, named Crf2 and used for the selection of single chain variable fragments (scFvs) by phage display, followed by characterization of these antibody fragments and the specific detection of *A. fumigatus*.

## Results

### Isolation and characterization of Crf2

**Figure 1:**
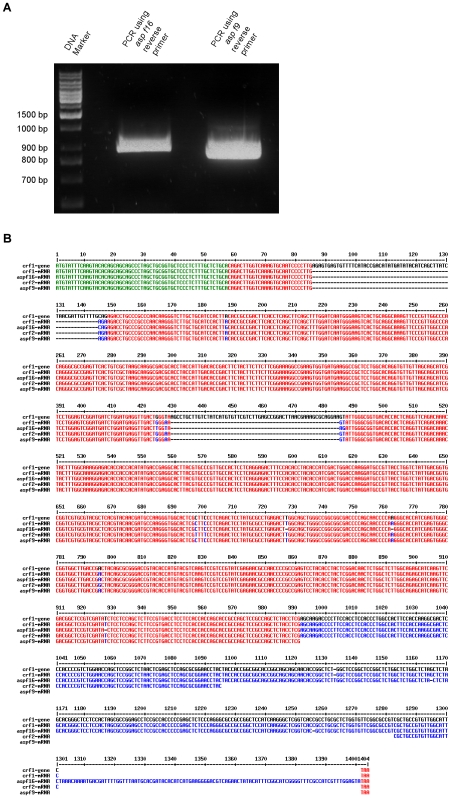
Isolation and analysis of *crf2*. A Isolation of *crf2*. PCR products derived from cDNA using primer sets for the amplification of *asp f16* (resulting in the amplification of *crf2*) or *asp f9* were separated by 1% agarose gel electrophoresis. B Alignments of the nucleotide sequences of *crf1* gene (NCBI: NC_007194.1, CADRE: AFUA_6G08510), mRNA of *crf1* (NCBI: AY169706), mRNA of *crf2*, mRNA of *asp f9* (NCBI: AJ223327) and mRNA of *asp f16* (NCBI: AF062651). The primer sequences for the amplification of *asp f9* and *asp f16* are underlined. The *asp f16* reverse primer1 binds directly downstream of the *crf1* gene stop codon. If the *crf1* gene sequence is identical with all mRNA sequences the sequences are red. The *crf1* gene sequence is in black and the mRNA sequences are in blue, if differences between the *crf1* gene sequence and any mRNA sequence. The leader sequences are marked in green.

An *A. fumigatus* cultivation derived from bronchoalveolar lavage material of a human patient with proven invasive aspergillosis (IA) was used as a source for mRNA isolation and reverse transcription (RT-) PCR. By using *asp f16* (NCBI [www.ncbi.nlm.nih.gov]: AF062651) or *crf1* (NCBI: NC_007194.1) (CADRE [www.cadre-genomes.org.uk] [30]: AFUA_6G08510) specific PCR-primer sets, two PCR products of 822 and 948 bp were obtained instead of the expected product of about 1134 bp representing full-length *asp f16*. The first PCR product encoded *asp f9* with one point mutation (aa position 218 M>V) and the second did not represent any known gene product (Fig. 1).

**Figure 2:**
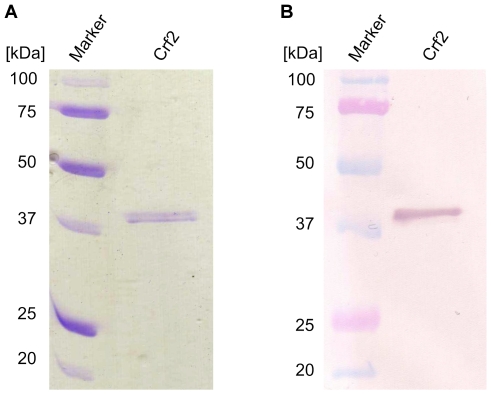
Production and purification of Crf2. IMAC and IEC purified Crf2 was separated on a reducing 12% SDS-PAGE. A Coomassie staining. B the recombinant Crf was detected with mouse anti-his tag (1∶10,000) and goat anti-mouse IgG (Fc specific) AP conjugate (1∶10,000).

Cloning and sequence analysis of all obtained amplification products confirmed the absence of full-length *asp f16*, but identified a novel variant, which we named *crf2* due to its partial similarity to the published *crf1* [31]. This variant encodes aa 1–327 of Crf1 (XP_752985), which are directly followed by aa 390–395 of Crf1 and a stop codon (Fig. 2).

### Production of Crf2

The Crf2 encoding cDNA was cloned into a pET21a(+) plasmid (Invitrogen, Karlsruhe, Germany) using *E. coli* XL-1 Blue MRF' (Stratagene, Amsterdam, Netherland) cells. Crf2 protein expression was performed in BLR(DE3) (Invitrogen) cells and purified by immobilized metal affinity chromatography (IMAC) followed by ion exchange chromatography (IEC). The production and purification was analysed by SDS-PAGE followed by immunoblot (Fig. 2). The purified Crf2 protein preparation did not contain contaminations detectable by Coomassie stained SDS-PAGE (Fig. 2A). Immunoblots did not indicate breakdown products (Fig. 2B).

### Verification of the expression of Crf2 during *A. fumigatus* infections

**Figure 3:**
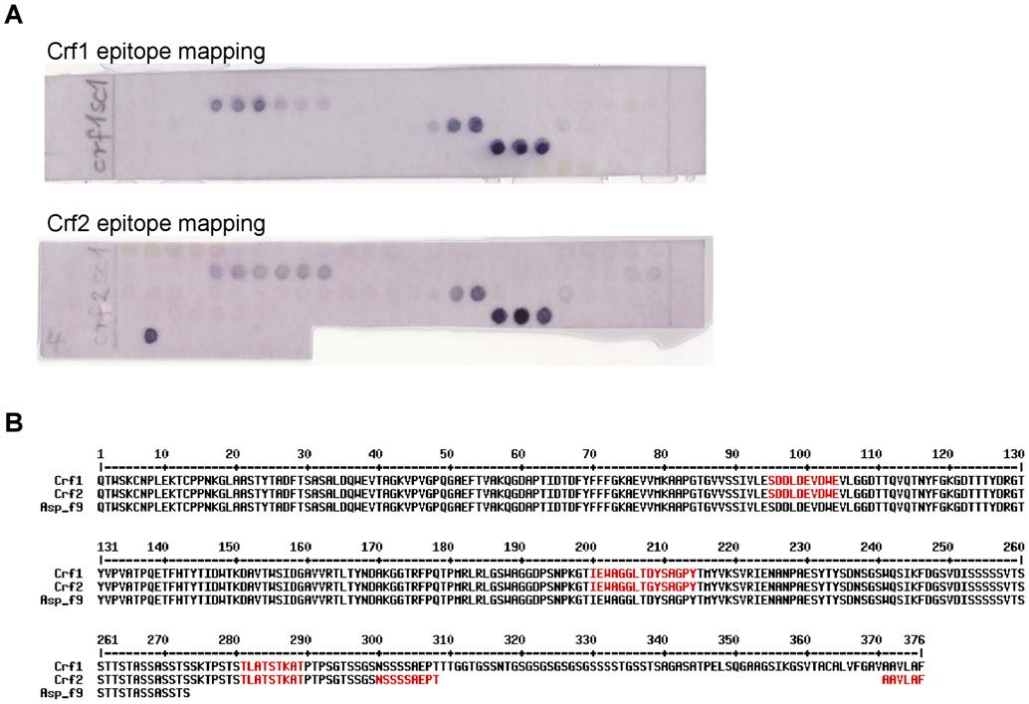
Epitope mapping of serum of mice infected with *A. fumigatus* on Crf1 and Crf2. A Epitope mapping membrane (15mer oligopeptide, 3 aminoacid overlap) stained with mouse serum (1∶400). The bound mouse antibodies were detected with goat anti-mouse IgG Fc specific AP conjugated (1∶2000). The stained Crf2 specific epitope was marked with a square. B Aligment of Crf1, Crf2 and Asp f9. The recognized epitopes of Crf1 and Crf2 are marked in red (the shorter polypeptide Asp f9 is shown for comparison, Asp f16 is not shown due to a frameshift resulting in a divergent amino acid sequence compared to Crf1, Crf2 and Asp f9).

To verify that Crf2 is expressed by *A. fumigatus* during infection, mice were infected with *A. fumigatus* strain D141, isolated from a patient with an aspergillom [32], and the serum was used for an epitope mapping on Crf1 and Crf2 (Fig. 3A). Three epitopes were detected on both proteins (Fig. 3B). One continuous epitope (NSSAEPTAAVLAF) identified on Crf2 is specific for Crf2 and not part of Crf1, Asp f9 (also shown in the alignment) or Asp f16 (not shown in the aligment, due to frameshifts compared to Crf1, Crf2 and Asp f9). Thus, Crf2 was expressed by two different clinical isolates during infection.

An epitope mapping was also performed for Asp f16. Here, only epitopes were detected which are in the amino acid segments of Asp f16 identical to Crf1. No epitopes were detected where Asp f16 is different to Crf1 (data not show).

### Immunization of macaques and generation of an immune antibody gene library

**Figure 4:**
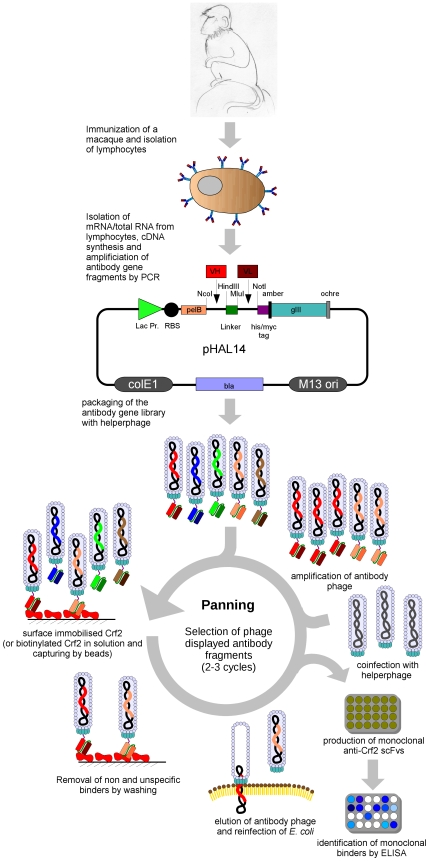
Schematic overview about of the construction of macaque immune libraries, selection and screening of antibodies.

**Figure 5:**
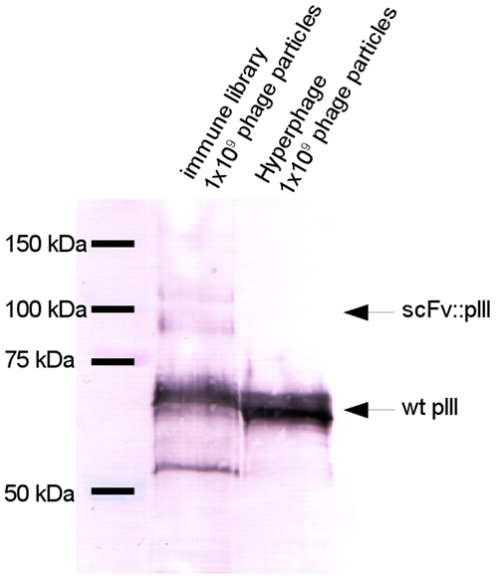
Immunoblot of the Hyperphage packaged macaque library. 1×10^9^ or 5×10^9^ scFv phage particles or 1×10^9^ Hyperphage particles per lane were separated on a reducing 10% SDS-PAGE, followed by Western blot and detection of wildtype pIII or scFv::pIII fusion using mouse mAb anti-pIII (1∶2000) and goat anti-mouse HRP (1∶5,000).

The procedure of immunization, library cloning, selection of antibodies and screening is given in figure 4. A male macaque was immunized with recombinant Crf2. After four antigen injections, the Crf2 specific antibody titre measured by ELISA was equal to 1∶256,000. Antibody fragment PCR products amplified from the obtained bone marrow cDNA were cloned in pGemT, resulting in a VH sub-library consisting of 6.2×10^5^ independent clones and a VL sub-library of 1.6×10^5^ clones. The scFv libraries were constructed in pHAL14 by two consecutive cloning steps, starting with the VH gene fragments, followed by the VL fragmenst. The final scFv antibody gene library consisted of 1.5×10^7^ independent clones with 80% full size inserts. The anti-Crf2 immune library was packaged with Hyperphage and showed a sufficient scFv surface presentation on phage, as determined by SDS-PAGE, Western blot and anti-pIII immunostain (Fig. 5). Two scFv::pIII fusion proteins were detected, as described before [34], [33].

### Isolation of recombinant antibodies against Crf2

In addition to the anti-Crf2 macaque immune library, the human naive antibody gene library HAL4/7 [35] was used for the isolation of recombinant antibodies against the recombinant Crf2 protein. Both antibody gene libraries were screened with two different panning strategies. In the first panning strategy, immunostripes were coated with recombinant Crf2. In the second strategy, the library was preincubated with *in vivo* biotinylated recombinant Crf2 in solution which was captured using streptavidin beads. After panning, individual clones were isolated and monoclonal scFv antibody fragments were produced in MTPs. To identify binders, the culture supernatants of the scFv clones were directly used for antigen ELISA using either Crf2 directly coated on Maxisorp microtitre plates (Nunc), when clones were isolated by the first panning strategy, or biotinylated Crf2 bound on streptavidin microtitre plates (Nunc) when the second panning strategy was used. Afterwards, monoclonal binders were analysed by DNA sequencing to exclude identical clones. As expected, more unique binders were isolated from the macaque immune library as from the naive human antibody gene library. In comparison to the panning strategy with biotinylated Crf2 in solution, the panning strategy using Crf2 coated immunostripes (microtitre wells) resulted in more primary hits but the number of unique scFv clones was the same (Table 1).

**Table 1:** Panning results on Crf2.

Antibody gene library (panning strategy)	Screened monoclonal scFvs^1^	Monoclonal binders^2^	Unique monoclonal binders^3^
**HAL 4/7**			
immunustripe	196	11	5
In solution	184	2	1
**macaque immune library**			
immunostripe	188	67	4
In solution	184	6	6

1 Total number of scFv screened after panning.

2 Monoclonal scFvs binding to Crf2 in ELISA.

3 Unique binders identified by DNA sequencing.

The unique scFvs were analysed for their variable immunoglobuline gene segments using VBASE2 (www.vbase2.org) [36] (Table 2). The selected scFvs isolated from the naive antibody gene library were very different in respect of the VH and VL genes. In contrast, the scFv isolated from the immune antibody gene library had only two different VH genes associated with different VL genes. Interestingly, the VL gene of MS130i-IIIC3 was most similiar to a murine VL germline gene.

**Table 2:** Classification of the selected anti-Crf2 scFv gene fragments with human gene segments according to VBASE2.

scFv clone	antibody gene library	VH			VL		germinality index (VH/VL)
		HV	D	HJ	LV	LJ	
DOH12.2-IIIA11	HAL4/7	IGHV3-23*01	IGHD5-12*01	IGHJ4*02	IGKV2-30*01	IGKJ1*01	0.97/0.93
DOH12.2-IIIB8	HAL4/7	IGHV3-30*01	IGHD2-2*02	IGHJ3*02	IGLV1-40*01	IGLJ3*01	0.97/0.94
DOH12.2-IIID10	HAL4/7	IGHV3-30*01	IGHD2-2*02inv	IGHJ3*02	IGLV1-40*01	IGLJ3*01	0.96/0.93
DOH12.2-IIID11	HAL4/7	IGHV1-46*01	IGHD5-5*01	IGHJ6*02	IGLV7-43*01	IGLJ3*01	0.96/0.98
DOH12.2-IIIE11	HAL4/7	IGHV3-30*03	IGHD6-19*01	IGHJ4*02	IGLV2-8*01	IGLJ3*01	0.96/0.98
MS130h-IIIC2	HAL4/7	IGHV1-2*02	IGHD3-10*01	IGHJ2*01	IGLV3-1*01	IGLJ3*01	0.94/0.93
MS112-IIA1	immune	IGHV4	IGHD2-2*01	IGHJ5*01	IGKV1-9*01	IGKJ2*01	0.94/0.93
MS112-IIB1	immune	IGHV4	IGHD3-10*02	IGHJ1*01	IGKV1-39*01	IGKJ2*01	0.79/0.94
MS112-IB10	immune	IGHV5-51*01	IGHD5-5*01	IGHJ6*02	IGKV1-27*01	IGKJ4*01	0.92/0.96
MS112-IIF5	immune	IGHV5-51*01	IGHD3-22*01	IGHJ4*02	IGKV1-39*01	IGKJ1*01	0.91/0.91
MS130i-IIIC3	immune	IGHV5-51*01	IGHD3-22*01	IGHJ4*02	IGKV4-74*01	jk1	0.9/0.86
MS130i-IIIG3	immune	IGHV5-51*01	IGHD3-22*01	IGHJ4*02	IGKV1-9*01	IGKJ3*01	0.88/0.88
MS130i-IIB6	immune	IGHV5-51*01	IGHD6-19*01	IGHJ4*02	IGKV1-5*03	IGKJ2*01	0.92/0.92
MS130i-IID7	immune	IGHV5-51*01	IGHD3-22*01	IGHJ4*02	IGKV1-39*01	IGKJ1*01	0.90/0.89
MS130i-IIF6	immune	IGHV5-51*01	IGHD3-22*01	IGHJ4*02	IGKV1-9*01	IGKJ4*01	0.89/0.92
MS130i-IIF4	immune	IGHV5-51*01	IGHD7-27*01	IGHJ4*02	IGKV1	IGKJ1*01	0,93/0,94

Abbreviations: HV: V (variable) gene segments of the heavy chain; D: D (diversity) gene segment; HJ: J (joining) gene segment of the heavy chain; LV: V gene segment of the light chain; LJ: J gene segment of the light chain; HAL4/7: human, naive antibody gene library; immune library: immune library derived from Crf2 immunized macaque. The germinality index describes the similarity of the anti-Crf2 scFvs to the most similiar human germline genes identified by VBASE2 (www.vbase2.org) indicated by percental identity of the VH or VL framework region.

The similarity to human germline genes of the VH and VL genes (germinality index) of the anti-Crf2 scFvs was calculated according to Pelat et al. [37] indicated by percental identity of the VH or VL framework region to the most similar human germline gene identified by VBASE2 (Table 2). As expected, the VH and VL of the scFvs derived from the human naive antibody gene library HAL4/7 had a higher identity to human germline genes than the V regions of the scFvs from the macaque immune antibody gene library.

### Immunoblot analysis of the anti-Crf2 scFvs

The binding of the selected unique anti-Crf2 scFvs to denatured Crf2 was analysed by immunoblot. Crf2 was separated by SDS-PAGE, followed by immunoblot using anti-Crf2 scFv and the corresponding secondary antibodies (Table 3). Here, the panning strategies yielded different results. All Crf2 specific scFvs obtained by panning in immunostripes bound only to denatured Crf2, i. e. to linear epitopes, whereas all scFvs selected by panning in solution bound only to conformational epitopes. These results were independent from the origin of the scFv from either an immune or naive antibody gene library.

**Table 3:** Properties of the Crf2 specific scFv antibody clones.

scFv clone	Antibody gene library	Panning protocol	Epitope^1^	Affinity KD [M]	Serum stability^2^ [days]	Antigen detection limit^3^ [ng/mL]	Antigen binding analysed by fluorescence microscopy		
							*Aspergillus fumigatus*	*A. terreus, nidulans, flavus, clavatus*	*Candida albicans*
DOH12.2-IIIA11	HAL4/7	immunostripe	linear	n.d.	n.d.	n.d.	no	n.d.	n.d.
DOH12.2-IIIB8	HAL4/7	immunostripe	linear	n.d.	n.d.	n.d.	no	n.d.	n.d.
DOH12.2-IIID10	HAL4/7	immunostripe	linear	n.d.	n.d.	n.d.	no	n.d.	n.d.
DOH12.2-IIID11	HAL4/7	immunostripe	linear	n.d.	n.d.	n.d.	no	n.d.	n.d.
DOH12.2-IIIE11	HAL4/7	immunostripe	linear	n.d.	n.d.	n.d.	no	n.d.	n.d.
MS130h-IIIC2	HAL4/7	solution	conformational	1.4×10^-8^	>27/>27	75	yes	no	no
MS112-IIA1	immune library	immunostripe	linear	1.5×10^-9^	<1/9-12	2	yes	no	no
MS112-IIB1	immune library	immunostripe	linear	3.9×10^-10^	<1/>27	5	yes	no	no
MS112-IB10	immune library	immunostripe	linear	n.d.	n.d.	n.d.	no	no	no
MS112-IIF5	immune library	immunostripe	linear	n.d.	n.d.	n.d.	n.d.	n.d.	n.d.
MS130i-IIIC3	immune library	solution	conformational	9.6×10^-10^	<1/9-12	150	yes	no	no
MS130i-IIIG3	immune library	solution	conformational	4.1×10^-9^	<1/<1	>600	yes	no	no
MS130i-IIB6	immune library	solution	conformational	2.5×10^-7^	<1/<1	>600	yes	no	no
MS130i-IID7	immune library	solution	conformational	5.4×10^-8^	3-6/1	>600	yes	no	no
MS130i-IIF6	immune library	solution	conformational	1×10^-8^	<1/<1	>600	n.d.	n.d.	n.d.
MS130i-IIF4	immune library	solution	conformational	4.1×10^-7^	3/3	>600	n.d.	n.d.	n.d.

Abbreviations: n.d. - not determined,

1 Epitopes were concluded from scFv binding to denatured Crf2 in Western immunoblots (linear epitope) or exclusive binding to native antigen on A. fumigatus in immunofluorescence microscopy (conformational epitope).

2 Serum half life of the scFvs were determined by incubation in PBS supplemented with human serum at 37°C.

3 Antigen detection limit of the scFv clones was measured in ELISA. Streptavidin was used for capturing biotinylated Crf2 without denaturation. Detection was performed with the scFv clones.

### Binding of the scFv antibodies to A. fumigatus hyphae

The binding of the selected antibody fragments to native Crf2 on *A. fumigatus* hyphae and spores was analysed by immunofluorescence microscopy. For this purpose, the scFvs were recloned into the mammalia expression vector pCMV-hIgG1-Fc-XP [33] to convert them into the IgG-like bivalent scFv-Fc antibody format. The scFv-Fc fusion proteins were transiently produced in HEK293T cells and purified by protein A affinity chromatography.

**Figure 6:**
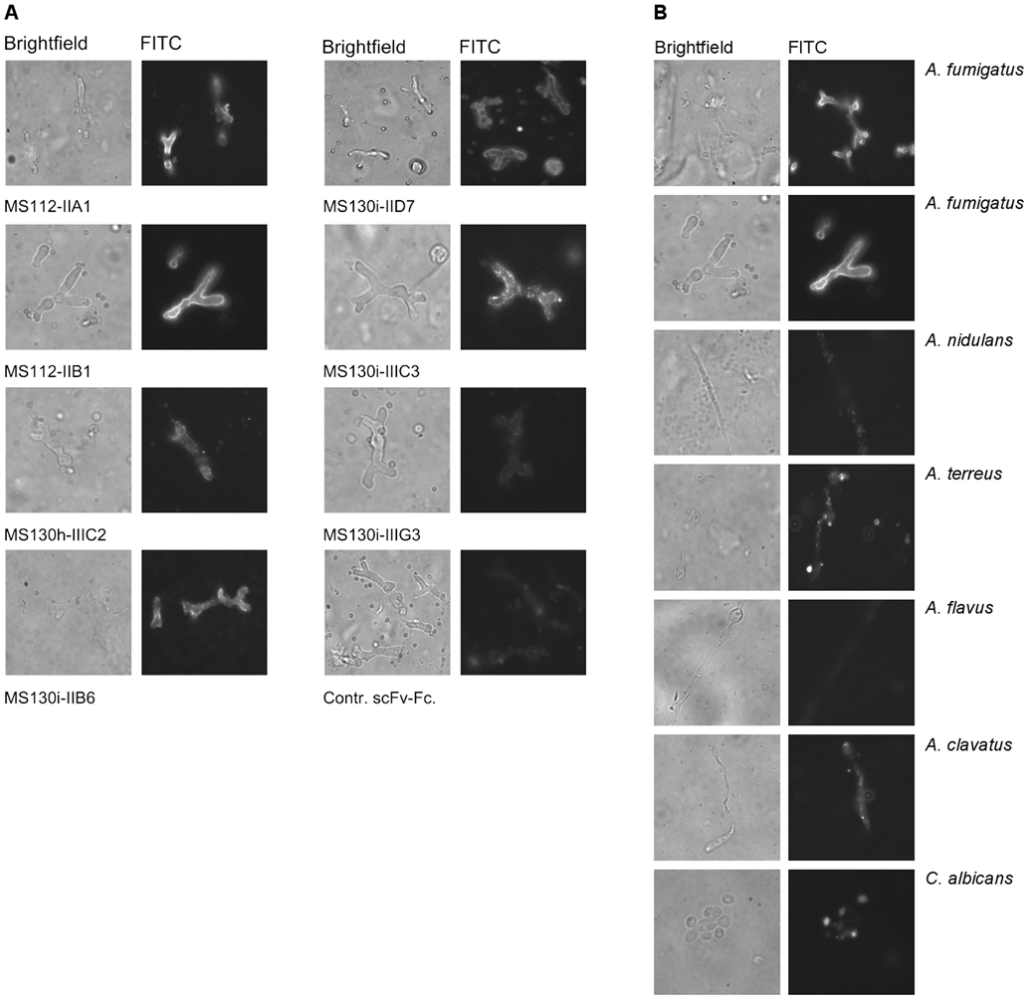
Fluorescence microscopy using anti-Crf2 scFv-Fc fusion proteins. A Binding of anti-Crf2 scFv-Fc fusion proteins to *A. fumigatus* (left panel brightfield, right panel FITC). B Binding of MS112-IIB1 to different *Aspergillus* strains (*A. fumigatus*, *A. nidulans*, *A. terreus*, *A. flavus*, *A. clavatus*) and *Candida albicans*. The staining was performed using 2 µg/mL scFv-Fc fusion protein and goat anti-human IgG (Fc specific) conjugated with Alexa 488 (1∶500).

One day after germination, spores and growing hyphae of *A. fumigatus*, other *Aspergillus* species (*A. terreus*, *A. nidulans*, *A. flavus*, *A. clavatus*) and the human pathogen *Candida albicans* were immobilized on poly L-lysine coated cover slips, fixed and stained with anti-Crf2 scFv-Fc fusion proteins and the corresponding secondary antibodies and examined by brightfield and fluorescence microscopy. The staining of *A. fumigatus* for all analysed scFv-Fc clones (Fig. 6A) revealed a pattern on the cell surface on the cell surface, restricted to the growing part of the hyphae. Spores were not stained. Seven of the analysed scFv-Fc fusion proteins bound specifically to *A. fumigatus* and did not bind any other *Aspergillus* species or *C. albicans* (Fig. 6B, shown for MS112-IIB1). These antibody clones were not only able to bind Crf2 or an other *crf1* gene product in its native conformation on cells but also allowed the discrimination of *A. fumigatus* from related *Aspergillus* species. Several of the antibodies identified to recognize linear epitopes bound to native Crf2 on cells.

### Characterization of anti-Crf2 scFvs by titration ELISA

**Figure 7:**
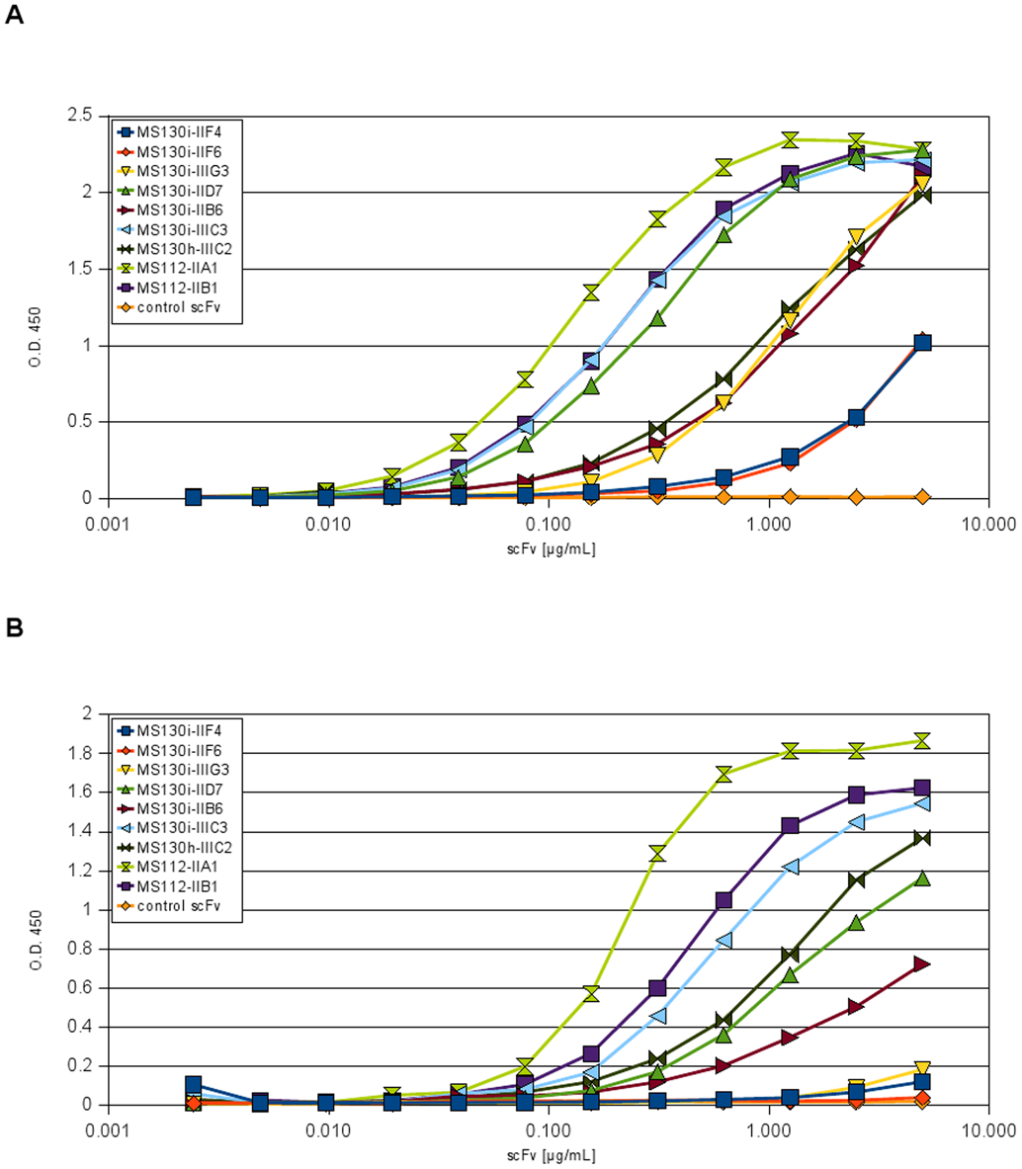
Analysis of the antigen binding by titration ELISA to analyse the binding of the anti-Crf2 scFvs to differently immobilized recombinant Crf2. 400 ng biotinylated Crf2 was immobilized on streptavidin microtitre plate to keep the native folding (A) or directly coated onto Maxisorp plates resulting in partial denaturation of Crf2 (B). A dilution series of scFvs were used for Crf2 detection. The bound scFvs were detected with mouse anti-myc (1∶1,000) and goat anti-mouse IgG (Fab specific) HRP conjugate (1∶10,000).

The scFv clones which bound to native Crf2 on *A. fumigatus* were further characterized by titration ELISA on biotinylated Crf2 coated on streptavidin plates (Fig. 7A). The scFv clones MS112-IIA1, MS112-IIBI, MS130i-IIIC3 and MS130i-IID7 showed half maximal antigen binding in low concentrations of ∼100–200 ng/mL and the clones MS130h-IIIC2, MS130i-IIIG3 and MS130i-IIB6 at ∼1 µg/mL, respectively. The antibody fragments MS130i-IIF4 and MS130iIIF6 bound only in high concentrations around 5 µg/mL. If the antigen was directly coated to the plastic surface of Maxisorp plates, a higher scFv concentration was required to reach half maximal antigen binding in the ELISA. Moreover, three scFv clones (MS-130i-IIIG3, MS130i-IIF4, MS130i-IIF6) showed nearly no binding on Crf2 when it was directly coated in Maxisorp plates (Fig. 7B).

### Determination of the affinity of anti-Crf2 scFvs by surface plasmon resonance (SPR)

**Figure 8:**
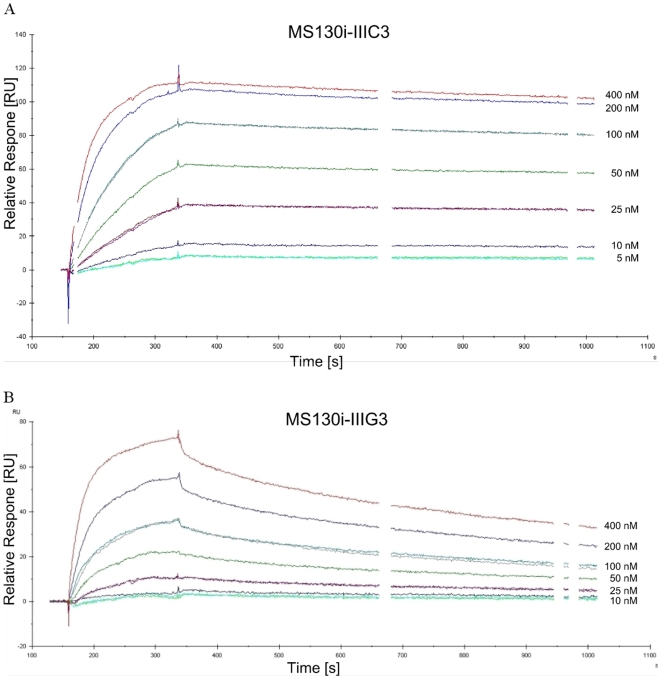
Determination of the affinity of the anti-Crf2 scFvs by surface plasmon resonance. SPR analyses of scFv MS130i-IIIC3 (A), scFv MS130i-IIIG3 (B).

The affinity of the scFvs which bound to native Crf2 and all scFvs derived from panning in solution was determined by surface plasmon resonance. Biacore measurements for MS130i-IIIC3 and MS130i-IIIG3 are exemplarily given in Fig. 8 and all other results are summarized in Table 3. The scFvs MS112-IIB1 and MS130i-IIIC3 bound with subnanomolar affinity to Crf2. The other analysed scFvs bound with affinities between KD 10^−7^–10^−9^ M.

### Analysis of the stability of anti-Crf2 scFvs

**Figure 9:**
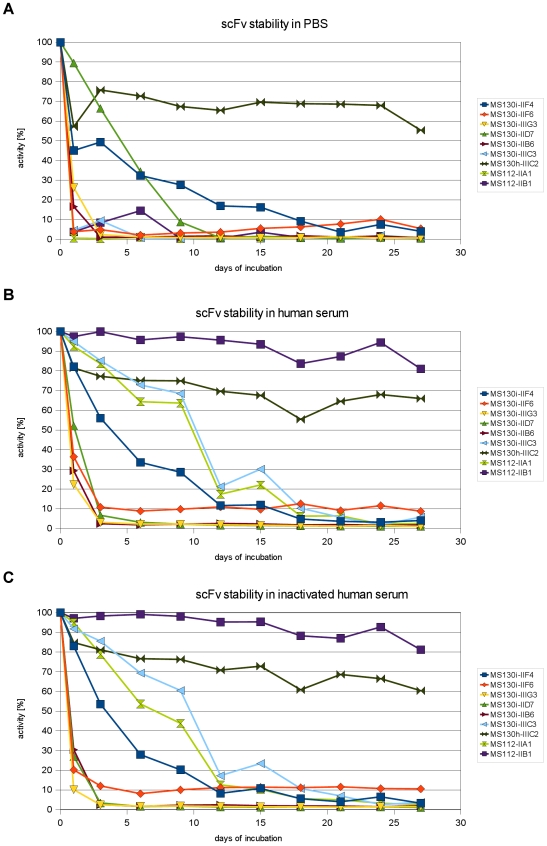
Analysis of the anti-Crf2 scFv stability by titration ELISA. Antigen: 400 ng biotinylated Crf2 coated on streptavidin microtitre plates. ScFvs were incubated in PBS (A), human serum (B) and inactivated human serum (C) for 0 to 27 days at 37°C and used for Crf2 detection. The scFvs were detected with mouse anti-myc (1∶1,000) and goat anti-mouse IgG HRP conjugated (1∶10,000).

The scFv clones which were specific for the native Crf2 were further analysed to evaluate their stability, an important feature of antibodies for potential diagnostic or therapeutic applications. Therefore, the scFvs were incubated in PBS, human serum or inactivated human serum respectively for 0 (control) to 27 days at 37°C. Crf2 binding activity was evaluated by antigen ELISA (Fig. 9, Table 3). The half life was longer for the scFv clones MS112-IIA1, MS112-IIB1 and MS130i-IIIC3 when incubated in serum compared to the incubation in PBS, whereas for the clone MS130i-IID7 the half life in PBS was better. The most stable scFv is MS130h-IIIC2 originated from the human naive antibody gene library with 60% Crf2 binding activity after 27 days of incubation at 37°C, independent of the incubation medium.

### Determination of the detection limit of recombinant Crf2 in spiked human serum

**Figure 10:**
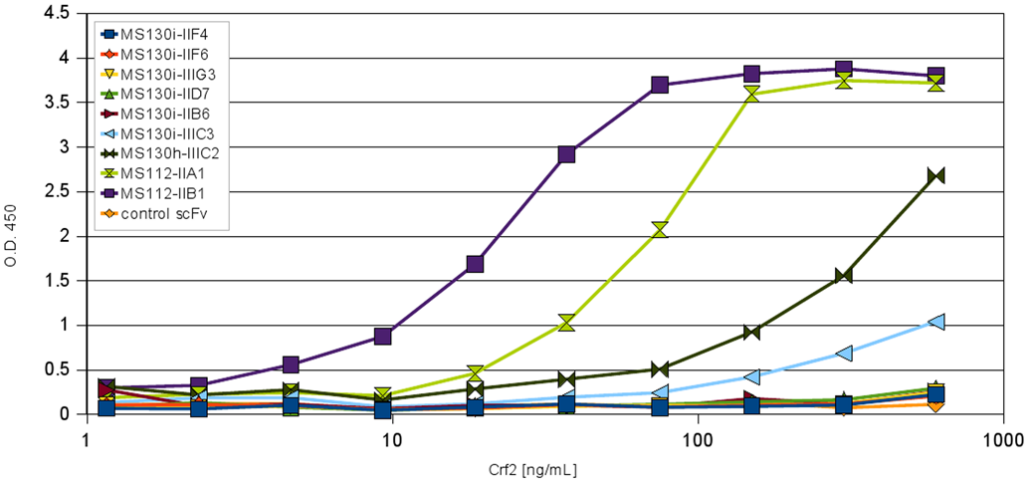
Detection of recombinant biotinylated Crf2 in human serum using anti-Crf2 scFvs. 400 ng anti-Crf2 scFvs were coated for capturing. Human serum was spiked with a dilution series of recombinant Crf2. The bound Crf2 was detected with 100 µL streptavidin HRP conjugate (1 µg/mL).

For diagnostic purposes, the detection limits of the scFvs which bound native Crf2 in solution were evaluated. Here, scFvs were coated to the plastic surface of Maxisorp microtitre plates to capture biotinylated Crf2 diluted in different concentrations in human serum (Fig. 10). Four scFvs (MS130i-IIIC3, MS130h-IIIC2, MS112-IIA1, MS112-IIB1) were able to detect Crf2 in serum and therefore are candidates for a future diagnostic assay. The estimated detection limits are given in Table 3. The scFv clones MS112-IIA1 and MS112-IIB1, which bound to linear epitopes, showed the best detection limit of 2 ng Crf2/mL or 5 ng Crf2/mL, respectively.

## Discussion

The specificity and sensitivity of IA - caused by *A. fumigatus* - assays has to be improved to allow optimal and early antifungal treatment with antimycotics [18], [5], [19]. Initially, we intended to generate recombinant antibodies against the well-characterized *A. fumigatus* antigens Asp f9 and Asp f16/Crf [22]–[24], [27]. Therefore, we tried to isolated the cDNA of the corresponding transcripts to obtain recombinant antigens for the generation of recombinant human antibodies against their gene products. Both *asp f9* and *asp f16* are splicing variants of the *crf* gene [22]. However, we were not able the isolate *asp f16* cDNA with specific primers from a clinical sample of a patient diagnosed with invasive infection of an *A. fumigatus* strain. Despite the fact that Asp f16 was successfully used as recombinant protein for active immunizations of mice which even protected them against IA [23], [28], the existence of *asp f16* as splicing variant is at least doubtful [29] as supported by epitope mapping results in this study. Instead of the *asp f16* cDNA, a related cDNA was isolated from the patient sample. The new transcript was named *crf2* according its parental gene *crf1* [31] which is also referred as *crf*. The new *crf2*, which is lacking a significant part of the 3′ end of *crf1*, is hypothesized to be a splicing variant of *crf1*. However, by bioinformatic analysis using Signal P [38] no splicing sites were found on these positions. Mice sera obtained after A. fumigatus infection bound to an epitope consisting of a sequence covering both ends of the potential splicing site, thus proving the existence of this gene product. Interestingly, this epitope is not part of Crf1, Asp f9 or Asp f16. Therefore, Crf2 was expressed by two different clinical isolates during infection.

In the further study, we focused on the generation of recombinant antibodies by antibody phage display against the novel *A. fumigatus* antigen Crf2. Antibody phage display is an *in vitro* technology to select recombinant antibodies against nearly any target for research, diagnostic and therapy [40]–[43], [39]. In this work, recombinant scFv antibody fragments were selected against Crf2 using two antibody gene libraries. Antibody fragments were isolated from the human naive antibody gene library HAL4/7 [33], [35] and an immune antibody gene library constructed from a macaque immunized with recombinant Crf2 protein. ScFvs were selected from both antibody gene libraries using two different panning protocols: First, the panning with recombinant Crf2 directly coated onto the plastic surface in immunostripes and second, the panning in solution with *in vivo* biotinylated recombinant Crf2 followed by pull down with streptavidin conjugated magnetic beads. Most Crf2 specific antibody fragments isolated from the human naive library bound to linear epitopes of Crf2, only one scFv antibody clone obtained by panning in solution recognized native Crf2. The macaque immune antibody gene library was expected to contain a larger number of Crf2-specific antibodies that also recognize native Crf2 because the macaque showed a high serum titre of Crf2-specific antibodies after immunization. The number of unique scFv antibody clones was slightly higher, but the impact of the panning strategy was even more significant. The panning using recombinant Crf2 directly coated on the plastic surface of immunostripes only led to the isolation of scFvs which bound to linear epitopes, whereas the strategy using *in vivo* biotinylated Crf2 in solution led to the isolation of scFvs which exclusively bound to conformational epitopes. The direct coating of proteins can lead to a partial denaturation [44], which can explain the selection of scFv antibody clones binding only to linear epitopes of Crf2. *In vivo* biotinylated Crf2 is not expected to be denatured in solution. The pull down with streptavidin coupled magnetic beads should not harm the conformation of Crf2. As expected, the selected macaque VHs or VLs showed a slightly lower identity to human germline genes as the human variable domains. For future therapeutic purposes, e.g. passive immunization, the macaque antibodies can be superhumanized if neccessary [37].

The selected scFvs were converted into the bivalent scFv-Fc antibody format and analyzed for their the binding to *A. fumigatus*, related *Aspergillus* species and the clinically relevant *C. albicans*. All analysed scFv-Fc proteins that recognized conformational epitopes were able to specifically bind to *A. fumigatus.* These antibodies bound obviously to the native conformation of Crf2 but binding to other Crf splicing variants is not excluded. In contrast to the scFv clones that recognized conformational Crf2 epitopes, only two of eight scFv-Fc proteins with the specificity for linear Crf2 epitopes also bound to *A. fumigatus*. The immunofluorescence staining showed that the anti-Crf2 scFv-Fc antibodies bound to the cell wall region of the growing hyphae. This is in accordance with publications showing that glycosylhydrolases/glycosyltranferases of the Crh and Gas family - Crf belongs to this group - are involved in the cell wall construction [22], [45], [46]. All generated scFv-Fcs bound specifically to *A. fumigatus* and not to other analysed pathogenic *Aspergillus* species or *C. albicans*. Therefore, these scFv-Fc promise to allow a specific histopathological identification of *A. fumigatus*. Immunofluorescence staining of histopathological samples of IA patients has already been described to be feasible for the detection of *A. fumigatus* [47].

In respect of a possible diagnostic application, the antibody clones that specifically bound to *A. fumigatus* were further characterized. Two different types of a dilution ELISA were performed and compared. In the first ELISA, *in vivo* biotinylated Crf2 was coupled to streptavidin coated microtitre plate wells, whereas in the second ELISA Crf2 was directly coated onto the plastic surface of Maxisorp microtitre plate wells. In general, scFv clones recognizing the native Crf2 bound better to streptavidin captured biotinylated Crf2 than to directly coated Crf2. Furthermore, three scFv clones which recognize conformational Crf2 epitopes did not bind to Crf2 coated on the plastic surface at all. This result also suggests that Crf2 becomes at least partially denatured after coating on plastic surfaces. This partial denaturation of proteins by immobilization [44] is only rarely described. It should be kept in mind when choosing the appropriate panning or assay protocols.

The affinity of the scFvs was determined by SPR. The scFv clone MS130h-IIIC2 from the naive human antibody gene library has an affinity of 10^−8^ M which is expected for isolated antibody fragments from large naive libraries by phage display [48]. In contrast, scFvs isolated from the macaque immune antibody gene library had affinities of up to 10^−10^ M.

The serum stability of the anti-Crf2 scFvs was very different among the individual clones and half life at 37°C ranged from less than one day to more than 27 days. The stability of one clone isolated from the human naïve antibody gene library and one clone of the macaque immune antibody gene library were exceptionally high. Here, the antigen binding activity only decreased by 20–30% after 27 days. In contrast, other publications showed half lives for scFvs at 37°C of less then one day to more than 6 days [49]–[51].

The human serum spiking experiment with a dilution series of biotinylated Crf2 established a detection limit to only 2 or 5 ng/mL recombinant Crf2. The results recommends these scFvs for a diagnostic detection of Crf2 in serum samples from patients with suspected IA. A Crf2 sandwich ELISA may improve and facilitate a reliable and early diagnosis of IA. Common *A. fumigatus* diagnosis assays like GM or BG assay are not specific for *A. fumigatus*, because other *Aspergillus* species and *Candida* are also detected. Furthermore, they are not very sensitive, e.g. the GM assay has a higher sensitivity for IA caused by other *Aspergillus* species and lead to many false positive results [5]. On the other hand the GM assay also detects IA caused by other *Aspergillus* species and other mycoses. Because of the side effect of antimycotics [10], [11] and the cost of the treatment of up to 20,000€ per patient [52], [53] a specific and reliable detection of *A. fumigatus* can improve the therapy and reduce costs.

The diagnosis and treatment of IA may benefit from the use of this novel antigen and the new antibody assay for its detection.

## Methods

### Ethics Statement

Human bronchoalveolar sample: No formal written approval of an ethical committee was needed for the human sample used as a template for the PCR since it fulfilled both of the following conditions: i) the sample was not taken for the purpose of scientific research but represented residual waste material from a routine test (otherwise to be discarded) ii) the sample(s) can not be linked to an individual person in any way.

Macaque immunization: The animal experiment was approved and performed according to following governmental french ethical guidelines: “Partie reglementaire du livre II du code rural (Titre I, chapitre IV, section 5, sous section 3:expérimentation sur l'animal)”, “Décret 87–848 du 19–10/1987 relatif aux expériences pratiquées sur les animaux vertébrés, modifié par le décret 2001/464 du 29/05/2001”, “Arrêté du 29 octobre 1990 relatif aux conditions de l'expérimentation animale pour le ministère de la défense” and “Instruction 844/DEF/DCSSA/AST/VET du 9 avril 1991 relative aux conditions de réalisation de l'expérimentation animale”

Mice infection: The experiment was approved by the appropriate national ethical board (Landesverwaltungsamt Halle, Halle (Saale), Germany/AZ: 509.42502/07-04.04.

### Cloning of *A. fumigatus* gene fragments and vectors

Bronchoalveolar material was isolated from the lung of patients with invasive aspergillosis (IA) by lavage and cultivated on *Aspergillus* minimal medium (AMM) [54] agar plates. The total RNA was isolated using Trizol (Invitrogen, Karlsruhe, Germany) according to the manufacturer's instructions. The cDNA was synthesized using SuperScript II (Invitrogen, Karlsruhe, Germany) and oligo(dT) primers following the manufacturer's instructions. The first strand cDNA was used as a template for the nested PCR using *asp f16* sequence specific forward primer1 5′ *a gca gcc cta gct gcg gtgc* 3′ and reverse primer1 5′ *ataagaacgatggagtagtc* 3′. For the amplification of *asp f16* the forward primer2 5′ *tataagctagc gca cag act tgg tca aag tgc aat* 3′ and reverse primer2 5′ *tattatgcggccgc gaa tgc caa cac ggc agc g* 3′ were used for the second PCR. For the isolation of *asp f9* the same forward and the reverse primer3 5′ *tattatgcggccgcgct cga ggt aga gct ggc gga* 3′ was used. The restriction sites for *Nhe*I and *Not*I of the *asp f16* and *asp f9* primers are underlined. The PCR products were cloned into the *Nhe*I/*Not*I site of the expression vector pET21a(+) (Novagen, Darmstadt, Germany) resulting in the vectors pET21a-Crf2 and pET21a-Aspf9a.

A bacterial expression vector for the *in vivo* biotinylation was constructed by hybridization of oligonucleotide primers encoding the gene fragment of the biotin acceptor domain (BAD) [55] and cloned into the *Xba*I/*Nhe*I site of pET21a-Crf2 vector resulting in the vector pET21a-Crf2-BAD. Transformations of XL1-Blue MRF' (Stratagene, Amsterdam, Netherlands) with these constructs were done by electroporation according to the manufacturers instructions. All affected regions of the constructs were comfirmed by DNA sequencing using an ABI Prism 310 sequencer.

### Immunization of mice

Female C57/Bl.6 mice (8–10 weeks old) used for the infection experiments were purchased from Harlan-Winkelmann (Borchen, Germany). Mice were intranasally inoculated with 30 µl of conidial suspension of Aspergillus strain D141, isolated from an aspergilloma patient [32], containing 5×10^6^ viable conidia. Blood was taken 7 days after infection from the retro-orbital sinus and serum was separated by centrifugation in BD Microtainer Tubes (BD Biosciences). After centrifugation the tubes were frozen at −20°C until tested by ELISA.

### Epitope Mapping

The protein sequence of Crf1, Crf2 and Asp f16 was divided into overlapping peptide fragments, each consisting of 15 amino acids, with an offset of three amino acids. This array of peptides was synthesized by the SPOT technique [56], [57] on an aminopegylated cellulose membrane (AIMS Scientific Products GmbH, Wolfenbüttel, Germany) as described previously [58]. Peptides are N-terminally acetylated and remain covalently attached to the membrane via their carboxy-terminus. The membrane bound peptide array was probed with serum of *A. fumigatus* infected mice for binding according to established procedures [58].

### Production of Crf2 in *E. coli*

For the production of Crf2 and biotinylated Crf2 the *E.coli* strain BLR(DE3) (Novagen) was transformed with the vector pET21a-Crf2 or cotransformed with pET21a-Crf2-BAD and the helper plasmid pRARE3 encoding the biotin ligase and rare tRNAs (Zoltan Konthur, Max Planck institute for molecular genetics, Berlin, Germany). Briefly, 1000 mL 2xTY [59]+100 µg/mL glucose+100 µg/mL ampicillin, respectively adding 1 mM biotin, were inoculated with an overnight culture yield to O.D._600_<0.1 and cultured at 37°C and 250 rpm. The induction was started by addition of 1 mM IPTG at O.D._600_ = 0.7 followed by cultivation for 5 h. Bacteria were harvested by centrifugation for 10 min at 4400×g at RT. Pellets were resuspended in 30 mL phosphate buffered saline (PBS) [59] and sonicated (Sonotrode MS72, Bandelin, Berlin, Germany). The resuspended cells were centrifuged for 10 min at 4400×g and the supernatant was filtrated using a 0.45 µm filter (Sartorius, Göttingen, Germany), before IMAC and IEC purification.

### Immobilized metal affinity chromatography (IMAC) and Ion exchange chromatography (IEC) purification of Crf2

Crf2 was purified from *E. coli* derived material by IMAC affinity chromatography using Äkta Prime (GE Healthcare, München, Germany) and a His Trap FF crude column (GE Healthcare) according to the manufacturer's instructions. The elution was performed using an imidazol gradient and the Crf2 containing elution fractions were dialysed over night against 5 L 20 mM TrisHCl, pH 8. Further purification by IEC was performed using a Resource Q (GE Healthcare) column, Crf2 was eluted using a NaCl gradient and dialysed against PBS.

### Immunization of macaques

A cynomolgus macaque (*Macaca fascicularis*) was immunized with Crf2, injected (200 µg per injection) subcutaneously with complete Freud adjuvant (Sigma, first boost) followed by incomplete Freud adjuvant. In total four boost were performed at day 19, 38, 66 and 77.

### Construction of the anti-Crf2 antibody gene library

Total bone marrow (around 5 ml) was sampled after the last boost to isolate RNA using Tri Reagent (Molecular Research Center Inc., Cincinnati, USA) according to the manufacturer's instructions. RNA was isolated, reverse transcribed with oligo(dT) into cDNA and amplified with primers specific for the DNA coding macaque Fd and VLκ as formerly published [60].

PCR products were cloned in the pGemT vector (Promega, Madison, Wisconsin) according to the manufacturer's instructions, yielding two antibody genes sub-libraries encoding the heavy chains (Fd fragment) or the kappa light chains. The pGemT cloned PCR products were reamplified using two oligucleotide primer sets to introduce restriction sites for library cloning. A set of macaque kappa oligonucleotide primers was used as forward oligonucleotide primers and reverse oligonucleotide primers [61]. The secondary PCRs were carried out for each forward oligonucleotide primers separately to keep the diversity. Each PCR was performed in a volume of 100 µl using 100 ng purified PCR reaction product of the pGemT cloned cDNA, 4 U Red Taq polymerase (Sigma, Hamburg, Germany), 200 µM dNTPs each and 200 nM of each oligonucleotide primer for 20 cycles (30 s 94°C, 30 s 57°C, 30 s 72°C), followed by 10 min 72°C. The PCR products were separated by 1,5% (w/v) agarose gel, cut out and purified using Nucleospin Extract II Kit (Macherey-Nagel, Düren, Germany) according to the manufacturer's instructions.

The construction of the library was done in two subsequent steps. First, the VH PCR products were cloned to pHAL14 as described [35], [34], [61] followed by a second cloning step to insert the VL PCR fragments. A total of 5 µg pHAL14 and 2 µg VH were digested using 50 U *Hind*III and 50 U *Nco*I (NEB, Frankfurt, Germany) in a 100 µl reaction volume for 2 h at 37°C. The enzyme reaction was inactived for 10 min at 65°C. Afterwards, 0.5 U calf intestinal phosphatase (MBI Fermentas) was added and incubated for further 30 min. This dephosphorylation step was repeated once. The vector was purified using the Nucleospin Extract II Kit. 270 ng VH were cloned into 1 µg of the dephosporylated pHAL14 using 1 U ligase (Promega, Mannheim, Germany) overnight at 16°C. The ligation reactions were precipitated with ethanol and sodium acetate and the pellet was washed twice with 70% ethanol. These reactions were electroporated (1.7 kV) in 25 µl XL1-Blue MRF' (Stratagene, Amsterdam, Netherlands). The transformed bacteria were plated onto 2xYT agar plates (25 cm petri dishes) supplemented with 100 µg/mL ampicillin and 100 mM glucose. The colonies were harvested by suspending in 40 mL 2xYT media with a Drigalsky spatula. Plasmids were isolated using the Nucleobond Plasmid Midi Kit (Macherey-Nagel) according to the manufactors instructions. 5 µg of each VH chain library as well as 1,5 µg of the VL fragments were digested using 50 U *Not*I and 50 U *Mul*I (NEB) in a 100 µl reaction volume overnight at 37°C. The following steps were performed as described for VH with the following modification: 250 ng of the digested and purified VL repertoire was used for ligation. In total 2 transformations were performed and pooled. The harvested bacteria representing the final antibody gene libraries were aliquoted and stored at −80°C.

### Selection of recombinant antibodies using immunostripes or magnetic beads

The selection of recombinant antibodies using microtitre plates/immunostripes is described by Hust et al. 2007 [39]. A total of 100 ng Crf2 was coated for the first panning round and second panning round per well and 10 ng Crf2 was coated for the last panning round.

For the panning in solution using streptavidin magnetic beads, two separate samples of 5 µL M280 beads (Dynal, Oslo, Norway) were preincubated in PBS with 1% (w/v) BSA and 1% (w/v) skim milk for 1.5 h at RT. All incubation steps were performed in an overhead shaker. The beads were captured using a magnetic separator (Dynal) and washed with PBS. One sample of the blocked beads was incubated with 1×10^11^ phage particles of the antibody gene libraries in 700 µL PBS with 1% (w/v) BSA and 1% (w/v) skim milk for 1.5 hours at RT and unspecific binder are removed using a magnetic separator. The supernatant with the residual library was incubated with 100 ng biotinylated Crf2 for 1.5 hours at RT followed by an incubation with the second sample of blocked streptavidin magnetic beads for another 20 min. The beads with the bound antibody phage were captured by pull down in a magnetic separator. The beads were washed 20 times with PBS. The elution of the bound scFv phage particles with trypsin and the reamplification of the scFv phage were done as described for panning in MTPs by Hust et al. 2007 [39].

### Enzyme linked immunosorbent assay (ELISA)

For the detection of anti-Crf2 antibodies by ELISA, recombinant Crf2 antigen was coated to 96 well microtitre plates (Maxisorp, Nunc) in PBS overnight at 4°C. To evaluate effects caused by partial denaturation of the antigen, biotinylated Crf2 was bound to 96 well streptavidin microtitre plates (Streptavidin F96 Clear, Nunc) in a parallel approach. To detect Crf2 antigen by ELISA, purified Crf2-specific scFvs were coated to Maxisorp plates in PBS at 4°C overnight. After coating, the wells were washed three times with PBST and blocked with 2% (w/v) skim milk powder in PBST (2%M-PBST) for 1.5 h at RT, followed by three washing steps with PBST. For the antigen ELISA soluble scFvs were diluted in 100 µL 2%M-PBST and incubated in the Crf2 coated plates for 1.5 h at RT followed by three times PBST washing cycles. For the antigen capture ELISA, the biotinylated Crf2 antigen was diluted in 100 µL 2%M-PBST and incubated with the capture antibody for 1.5 h followed by three washing steps with PBST. Bound scFvs were detected with the murine mAb 9E10 which recognizes the c-terminal c-myc tag and a goat anti-mouse Ab conjugated with horseradish peroxidase (HRP) (Sigma; 1∶10,000). Captured biotinylated Crf2 was detected with 1 µg/mL streptavidin conjugated with HRP. The visualization was performed with TMB (3,3′,5,5′-tetramethylbenzidine) as substrate and the staining reaction was stopped by adding 100 µl 1 N sulphuric acid. Absorbance at 450 nm was measured by using a SUNRISE™ microtitre plate reader (Tecan, Crailsheim, Germany).

### SDS-PAGE and Immunoblot

Crf2 or scFv proteins were analysed by 12% SDS-PAGE. Protein gels were stained with coomassie blue or blotted onto a PVDF membrane. The membrane was blocked with 2%M-PBST for 1 h at RT. The detection of Crf2 was performed using mouse anti-his tag (1∶5,000), the detection of scFvs was performed using mouse mAb 9E10 (anti-myc tag) (1∶500) and detected with a goat anti-mouse antibody conjugated to AP (Sigma, Taufkirchen, Germany) (1∶10,000). The staining was performed using NBT/BCIP substrate.

### Cloning and production of scFv-Fc fusion proteins

The recloning of anti-Crf2 scFvs into pCMV-hIgG1-Fc-XP and the transient production in HEK293T was performed as described before [33].

### Cultivation of fungi and immunofluorescence microscopy

*A. fumigatus* (clinical isolate D141) [32] and *A. flavus* (DSM818, Deutsche Stammsammlung für Mikroorganismen DSMZ, Braunschweig, Germany) were cultivated in potato dextrose medium according to DSM at 27°C. *Aspergillus terreus* (DSM1958) and *A. nidulans* (*Emericella nidulans*, DSM970) were cultivated in potato dextrose medium at 37°C. *Aspergillus clavatus* (DSM3410) was cultivated in Czapek-Dox medium according to DSM at 27°C and *C. albicans* (DSM3454) was cultivated in universal medium for yeasts (YM) according to DSM at 27°C. Subsequently, the spores were harvested and 4.5 mL RPMI 1640 medium [62] without fetal calf serum (FCS) was inoculated with ∼5×10^4^ spores. The spore solution was cultivated in 12 well plates (Greiner Bio-One, Essen, Germany) on poly L-lysine coated cover slips (Menzel-Gläser, Braunschweig, Germany) for one day at 37°C. The medium was removed and the the cover slips were washed three times with PBS.

The cover slips were blocked with 250 µL blocking solution (3% (w/v) BSA, 2% (w/v) skimmed milk powder and 0.05% (v/v) Tween 20 in PBS) for 1.5 h. A total of 250 µL scFv-Fc fusion protein with a final concentration of 2 µg/mL in blocking solution was incubated for 1.5 h and washed three times with PBS. The scFv-Fc fusion proteins were detected with 250 µL goat anti-human IgG (Fc specific) conjugated to Alexa 488 (1∶500 in blocking solution) for 1.5 h in the dark and washed three times with dH_2_O. The fungi were fixed with 10 µL Mowiol (Roth, Karlsruhe, Germany) and dryed overnight at RT in the dark.

An Axiovert 200 (Carl Zeiss, Germany) was used for fluorescence and bright field microscopy with 63×magnification and 1.0 gain for fluorescence or 0.002 gain for brightfield microscopy, respectively. All images were analyzed with the software LSM Image browser (Carl Zeiss, Germany).

### Surface plasmon resonance

The surface plasmon resonance was performed using Biacore 2000 according to the Biacore manual. Briefly, about 200 RU recombinant Crf2 were coupled in 10 mM sodium acetate buffer pH 4.0 on a CM5 chip. For reference, 200 RU BSA were coupled in 10 mM sodium acetate buffer pH 4.5. Serial dilutions of scFv (0 nM–400 nM) were measured at flow rate of 50 µL/min. The chip was regenerated with 100 mM glycine buffer pH 2.5. The data fitting was performed using 1∶1 Langmuir separate fitting algorithm of the Biaevalution software.

### ScFv stability analysis

The scFvs were aliquoted as triplicates in 100 µL (5 µg/mL in PBS or human serum) in 2 mL microtubes (Sarstedt, Nürnbrecht, Germany) and stored at −80°C. Every three days and every day for the last two samples, an aliquot was thawed and transferred into a 37°C incubator. All samples were analyzed at the same day in the same antigen ELISA using biotinylated Crf2 as antigen bound to streptavidin microtitre plates (Nunc). A total of 80 µL of the scFv solutions was analysed by antigen ELISA.
